# Assessment of Urinary Lead (Pb) and Essential Trace Elements in Autism Spectrum Disorder: a Case-Control Study Among Preschool Children in Malaysia

**DOI:** 10.1007/s12011-021-02654-w

**Published:** 2021-03-04

**Authors:** Mohd Shahrol Abd Wahil, Mohd Hasni Ja’afar, Zaleha Md Isa

**Affiliations:** Department of Community Health, Faculty of Medicine, National University of Malaysia, Kuala Lumpur, Malaysia

**Keywords:** Urinary, Pb, Trace elements, Autism, Children

## Abstract

Lead (Pb) is a heavy metal which is abundant in the environment and known to cause neurotoxicity in children even at minute concentration. However, the trace elements calcium (Ca), magnesium (Mg), zinc (Zn) and iron (Fe) are essential to children due to its protective effect on neurodevelopment. The primary objective of this study was to assess the role of Pb and trace elements in the development of autism spectrum disorder (ASD) among preschool children. A total of 81 ASD children and 74 typically developed (TD) children aged between 3 and 6 years participated in the study. Self-administered online questionnaires were completed by the parents. A first-morning urine sample was collected in a sterile polyethene urine container and assayed for Pb, Ca, Mg, Zn and Fe using an inductively coupled plasma mass spectrometry (ICP-MS). Comparisons between groups revealed that the urinary Pb, Mg, Zn and Fe levels in ASD children were significantly lower than TD children. The odds of ASD reduced significantly by 5.0% and 23.0% with an increment of every 1.0 μg/dL urinary Zn and Fe, respectively. Post interaction analysis showed that the odds of ASD reduced significantly by 11.0% and 0.1% with an increment of every 1.0 μg/dL urinary Zn and Pb, respectively. A significantly lower urinary Pb level in ASD children than TD children may be due to their poor detoxifying mechanism. Also, the significantly lower urinary Zn and Fe levels in ASD children may augment the neurotoxic effect of Pb.

## Introduction

Lead (Pb) is a naturally occurring, non-ferrous, heavy metallic element found in the earth’s crust. Despite being one of the most toxic pollutants, Pb has been used worldwide in various industries and consumers’ products due to its malleability and corrosion resistance [1]. Human exposure to Pb is inevitable due to the current industrial revolution, rapid urbanisation and economic development. It is worthy to note that Pb has potent and irreversible health effects on human. For instance, Pb could become a potential cofactor, initiator or promoter in many diseases, even at an extremely low concentration [2]. Therefore, frequent human exposure to Pb in many countries worldwide has become a global environmental health concern. Humans can be exposed to Pb through oral ingestion or inhalation of Pb-contaminated soil and dust [3].

Young children are more vulnerable to Pb exposure than adults, as they have unique physiological characteristics. Compared to an adult, children’s digestive system has a higher oral intake [3] and Pb absorption rate [4] which may increase further when they are fasting or lack of essential trace elements [5, 6]. Additionally, mouthing behaviours in young children may expose them to Pb [7]. Young children often have the habit of pica (persistent and compulsive cravings to eat non-food items) due to their innate curiosity and inability to differentiate between non-food items and food [8]. Apart from the digestive system, exposure to Pb can easily harm the children’s immature and developing nervous system [9].

There are many potential sources of Pb in the environment, including Pb mining and smelting, Pb-related industries (especially batteries and electronics), indoor and outdoor Pb-based paint, water piping and solder, domestic products (e.g. colour pencils, crayons, toys painted with Pb-based paint, Pb-glazed ceramic, cigarette, leaded petrol, cosmetic products and traditional remedies) and hobbies involving Pb (e.g. fishing, painting and collecting electronic devices) [4, 10–14]. Workmen in the Pb-related industries could contribute to “take-home contamination” by carrying Pb dust on their clothes, footwear, skin and other personal attires to their home [4, 15]. Children living in urban cities may be exposed to other sources of Pb pollution (e.g. soil and dust) and emissions produced by anthropogenic activities (e.g. road traffic, industries, construction and demolition) [2, 16]. They spread through the air rapidly within the environment, contaminate the food chain and eventually enter the human bodies [17]. Other associated risk factors of childhood Pb exposure are parents’ education levels, social status, children’s behaviours, habits, diet and nutritional status [18].

Childhood Pb toxicity is a preventable environmental disease that has long-lasting adverse health and behavioural effects. Children exposed to Pb are prone to experience irreversible morphological and molecular alterations of the nervous system [19–21]. It has been well established that Pb toxicity has various adverse effects on the central neurological function. Consequently, these effects increase the risk of a broad spectrum of developmental delays, intellectual and behavioural deficits, hyperactivity, social withdrawal, gross and fine motor performance deficits and decreased intelligence quotient (IQ) [22–27]. Additionally, these effects have been associated with higher Pb concentrations within hours following birth [28].

An acute high concentration of Pb toxicity in children rarely occurs nowadays due to the government’s law and legislation’s effectiveness in many developed countries to regulate and control Pb [29]. Examples of these control measures are phasing out Pb in petrol and household paint and reducing industrial emission, water Pb and other sources [29]. However, a chronic low Pb toxicity concentration is equally worrisome and more common in children [30]. Numerous neurocognitive and neurobehavioural effects were observed in children with blood Pb levels (BLLs) below 10.0 μg/dL [31–36]. The United States Centres for Disease Control and Prevention (CDC) initially defined the elevated BLLs as 10.0 μg/dL or greater [37, 38]. However, the CDC later lowered the value to 5.0 μg/dL in 2012 [39, 40]. Regardless of the cut-off BLL concentration, no Pb level can be considered safe due to its adverse effects on the children’s progressive neurodevelopment [41, 42].

Autism spectrum disorder (ASD) describes a range of neurodevelopmental disorders, as stated in the American Psychiatric Association’s Diagnostic and Statistical Manual of Mental Disorders 5th Edition (DSM-5) [43]. It is characterised by abnormal social behaviour, disinterest in communication and interaction, language disorders, repetitive and obsessive behaviours and narrowly focussed rigid interests [44]. Since its first description in 1943 with a prevalence of 4.5 cases per 10,000 children [45], there has been a large increase in ASD prevalence [46–50]. The cause and aetiology of ASD remain controversial. With no current consensus, investigators from various biomedical fields are studying multiple possible causes of ASD.

The role of environmental factors (e.g. neurotoxic heavy metal exposure) on the development of ASD cannot be overlooked. The broad spectrum of ASD also suggests that the disease’s phenotype heterogeneity may result from exposure to certain environmental agents, instead of primarily due to genetic disorder [51]. The neurotoxicity and heavy metal (including Pb) exposure have been associated with the cause of neurodevelopmental disorders [52]. Previous studies reported that Pb could damage the developing human brain, causing a broad spectrum of neurodevelopmental disorders [25, 36, 53]. Depending on the level of dose toxicity, the disorders could range from overt clinical manifestation (high-dose toxicity) to subclinical dysfunction (low-dose chronic toxicity) [25, 36, 53].

Essential trace elements, which include calcium (Ca), magnesium (Mg), zinc (Zn) and iron (Fe), play important roles in children’s normal brain development, neurotransmitter synthesis catabolism, cellular metabolic process, metabolism relevant to neurotransmitters and motor development [54–60]. Therefore, altered levels of these elements and their imbalance may lead to dysfunction of neurotransmitters. Neurotransmitter dysfunction has been observed in many medical conditions and diseases, including neurological and behavioural disorders [61–63]. However, less is known about the role of these elements in ASD development. It has been suggested that the essential trace elements caused the excitatory and inhibitory synapses in ASD to dysfunction [64].

Mainly, Ca is crucial for neurodevelopment and may provide preventive and therapeutic effects towards ASD by regulating synapse development and function [65]. Ca often binds rapidly to target proteins and subsequently regulate Ca channel function, generating Ca signalling [66–68]. Mg is the fourth most regulatory cation in the body that modulates gamma-aminobutyric acid (GABA) signalling [69, 70]. Mg also activates the copper-zinc superoxide dismutase (CuZn-SOD) and nitric oxide released from cells [71]. The CuZn-SOD and nitric oxide are important in brain development and functional well-being [71]. Zn is required to scaffold Pro-SAP/Shank proteins related to excitatory synapses, where lower Zn concentrations have been associated with ASD [72]. On the other hand, Fe is an essential element for DNA synthesis, gene expression, myelination, neurotransmission and mitochondrial electron transport [73]. These functions are crucial for the central nervous system. Therefore, Fe deficiency impairs the neurotransmitter processes, myelin formation and energy metabolism in the brain, which was thought to cause behavioural and cognitive developmental delays in children [74, 75].

Regular consumption of dairy products and milk formulas is beneficial for children’s health [76]. The foods contain a high nutritional value that provides high amounts of micro-nutrients, mainly Ca [76]. However, excessive Ca intake may cause nephrolithiasis, milk-alkali syndrome and interfere with the absorption of other essential trace elements, such as Mg, Zn and Fe [77]. Mg is widely distributed in leafy vegetables, legumes, nuts, seeds, whole grains, animal foods and beverages [77]. Zn sources can be found in either protein-rich plant (e.g. cereal grains and legumes) and animal protein food [78]. Phytate (e.g. wholegrain cereals, legumes, nuts and seeds) and dietary Ca were known to inhibit Zn absorption, while protein enhances Zn absorption [77]. There are three main dietary sources of Fe: (i) breast milk (where Fe is bound to lactoferrin), (ii) haem Fe (meat, poultry and fish) and (iii) non-haem Fe (e.g. spinach, lentils, pumpkin seeds, beans, nuts and fortified cereals) [79]. The absorption of non-haem Fe depends on the total net effect of factors enhancing Fe absorption, such as ascorbic acid and organic acids (e.g. meat, chicken, fish and seafood), fermented vegetables and fermented soy sauces and factors inhibiting Fe absorption (e.g. phytates and inositol phosphates, Fe-binding polyphenols, Ca, soy proteins and vegetable proteins) [77].

Pb exposure is common in urban cities in Malaysia. In 2000, the prevalence of children with BLLs above 10.0 μg/dL was 11.7% in urban Kuala Lumpur [80]. In 2015, a study revealed that 27.0% of children in urban Malacca had blood Pb levels above 10.0 μg/dL [81]. The Federal Territory of Kuala Lumpur is Malaysia’s national capital and forms the nation’s most populous urban region. It is the city’s increasingly global orientation and its implications for the wider urban region [82]. The city’s total land area is 243.70 km^2^ (24,221.05 ha), which is a hundred percent urban area. It had a population of 1,556,200 people in 2005, with an average population density of 64 persons per hectare [83]. The population increased to 1,790,000 people in 2018 [84]. Kuala Lumpur’s rapid urbanisation increases environmental pollution and exposes children to neurotoxic heavy metals, especially Pb. Therefore, Kuala Lumpur was the most appropriate location to conduct this study. To the best of our knowledge, no study has assessed urinary Pb and essential trace elements in ASD among preschool children in Malaysia. Therefore, the primary objective of this study was to assess the role of Pb and essential trace elements in ASD development among preschool children in Malaysia.

## Methodology

The current study protocol was approved by the National University of Malaysia (UKM) Research and Ethics Committee and the Medical Research and Ethics Committee of the Ministry of Health (MOH) Malaysia. All procedures were performed in accordance with the principles of the Declaration of Helsinki (1964) and later amendments. Participation was voluntary and informed written consents were obtained from the parents or legal caretakers before the study. This observational unmatched case-control study was conducted among the preschool children in Kuala Lumpur from January 15 until March 15, 2020. The study was completed before the first Movement Control Order (MCO) due to COVID-19 outbreak in Malaysia.

A total of 81 ASD children and 74 typically developed (TD) children were enrolled in the study. All children were Malaysian citizen aged between 3 and 6 years. Both group of children were randomly selected from the students’ name list with the schools’ permission. The ASD children were recruited from the national autism rehabilitation centre (GENIUS KURNIA), located in Sentul City, Kuala Lumpur. The centre is governed by the Ministry of Education (MOE) Malaysia. Clinical diagnosis of ASD was made by the paediatrician working in the government tertiary hospitals. The diagnosis was based on the DSM-5 criteria and the International Classification of Diseases-10 (ICD-10). The TD children in the control group were recruited from public preschools (age 4-6 years), namely TABIKA Department of Community Development (KEMAS) and public nurseries (age 2-4 years), namely TASKA KEMAS. The preschools and nurseries are located in Sentul City, Kuala Lumpur.

The two institutions were established and managed by the Early Childhood Education Division under the Ministry of Rural Development (MRD). The institutions were managed according to the National Preschool Standard Curriculum under the MOE, the National Early Childhood Care and Development Policy and the National PERMATA Curriculum [85]. The TD children were declared as “healthy”. They had no known characteristics of ASD, as verified by the paediatrician based on the Modified Checklist for Autism in Toddlers (M-CHAT) screening and the regular children health assessment during follow-up at 18 months and 36 months old. The following exclusion criteria were used for both groups: (i) congenital anomaly or syndrome, (ii) neurodevelopmental or neurobehavioural disorders, (iii) endocrine disorders, (iv) acute infectious, surgical and traumatic diseases and (v) currently on regular oral medications or infusion medications (chemotherapy) prescribed by specialists or on chelation therapy for heavy metal removal.

The researcher informed each participant’s parent (either father or mother) through phone calls, messages and emails to complete the self-administrated online questionnaire (Google Form). This method of gathering information online was preferred in this study due to several reasons: (i) easy access (through phone or computer), (ii) user-friendly, (iii) can be done at any time (especially for working parent who are busy during daytime), (iv) better data management (e.g. record keeping, confidentiality and data analysis) and (v) precautionary measure to the risk of COVID-19 transmission through closed contact during the COVID-19 outbreak in Malaysia. The researcher assisted the parent who had difficulty to complete the questionnaire through phone calls, messages and emails. The questionnaire was designed to elicit the information regarding the socio-demographic background of the parent and the child, the developmental milestone of the child, the risk factors that might indicate a predisposition to ASD (including pregnancy complications, preterm delivery, breastfeeding and family history of autism), the environmental exposure to Pb, the parental knowledge assessment on Pb and the dietary pattern of the child. The child’s anthropometric parameters (e.g. height and weight) were measured using calibrated digital weighing scales (Omron) that came with a height measurement stand. The researcher in the classroom recorded the measurements.

A first-morning urine sample was collected from each participant by the parent at home in a sterile polyethene urine container pre-treated with 20.0% nitric acid, HNO_3_ solution and rinsed twice with deionised water. Before the procedure, the parent was advised by the researcher regarding the correct technique of collecting urine sample: (i) the urine collection should be a clean catch, (ii) urine sample volume should range between 5.0 and 10.0 mL, (iii) the sterile polyethene urine container should not be contaminated with detergent, body soap or any foreign materials, (iv) the urine sample should not be added with water to avoid the dilutional effect and (v) proper closure of the urine container and the biohazard zip bag. The children were allowed to consume foods and drinks as usual. The urine samples were delivered by the parent to the researcher on the same day while they sent their children to the autism rehabilitation centre, preschool or nursery. The urine samples were labelled with code numbers and delivered to an accredited environmental laboratory, Faculty of Science and Technology, UKM, Bangi, Selangor within 24 h. The urine samples were stored at the temperature of −20.0 °C prior to the laboratory analysis.

In the laboratory, the urine samples were prepared by adding 1.0 mL of urine sample into 10.0 mL of 0.2% nitric acid (HNO_3_) solution with a ratio of 1:10. The preparation process was vital to allow the digestion process of organic matter in the urine sample. The prepared urine samples were then assayed for Pb and other essential trace elements (e.g. Ca, Mg, Zn and Fe) using the PerkinElmer SCIEX™ ELAN® 9000 inductively coupled plasma mass spectrometry (ICP-MS; PerkinElmer Inc., Shelton, CT 06484, USA). The detection limits using this operating system for each element were as follows: Pb 1.0–10.0 part per trillion (ppt), Ca 10.0–100.0 ppt, Mg 1.0–10.0 ppt, Zn 1.0–10.0 ppt and Fe 1.0–10.0 ppt. The system was calibrated using standard solutions prepared by the Universal Data Acquisition Standards Kit (Perkin Elmer Inc., Shelton, CT 06484, USA). Internal online standardisation was performed to assess the difference in matrix viscosity using 10.0 μg/L solutions of yttrium and rhodium Pure Single-Element Standard (Perkin Elmer Inc., Shelton, CT 06484, USA) [86].

The dataset from the questionnaire and the laboratory ICP-MS results were analysed using the IBM Statistical Package for Social Sciences (SPSS) software (version 22, IBM, Chicago, IL, USA). Prior to the statistical analysis, the data normality was explored graphically (based on histogram and Q-Q plot) and statistically (based on skewness, kurtosis and Shapiro-Wilks/Kolmogorov-Smirnov statistics). Frequency and percentage were calculated for each participant’s demographic parameters. The group differences in Pb levels (mean ± standard deviation) and other essential trace elements (Ca, Mg, Zn and Fe) were assessed either using the Student’s *t*-test (for normal distribution) or Mann-Whitney *U* test (for non-normal distribution). The potential associated risk factors and confounders (quantitative variables) were also assessed using one of the two methods mentioned above. The median, interquartile range (IQR), minimum and maximum of the analysed elements were also used as descriptive statistics. The categorical variables were calculated using the Chi-square test and presented in frequency and raw percentage. The magnitude of the correlation between element’s concentrations in the urine was analysed using the Pearson correlation test (for normal distribution) and by the Spearman rank correlation test (for non-normal distribution test). The receiver operating characteristic (ROC) analysis was performed as a comprehensive tool to assess the measured elements’ accuracy and choose the elements’ cut-off points. Simple and multiple logistic regression analyses were performed to assess the factors (independent variables included heavy metal, Pb) associated with ASD. The final prediction model allowed for an estimation of the effect (odds ratio) of the factors. A *p*-value of less than 0.05 was regarded as statistically significant in this study.

## Results

**Fig. 1:**
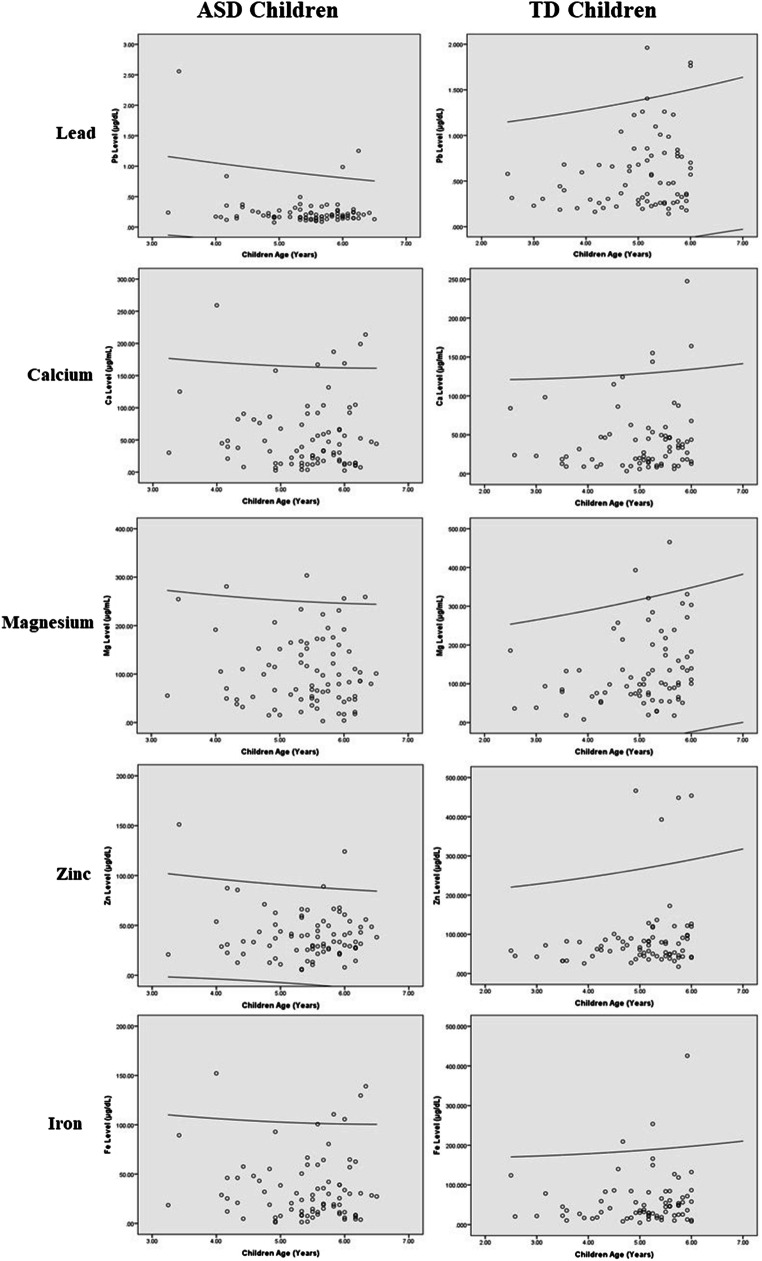
Comparison of concentration level of urinary Pb and essential trace elements (Ca, Mg, Zn and Fe) between ASD children and TD children according to children’s age (in months). The line represents 95.0% confidence interval (CI) of concentration level

Figure 1 shows a comparison of the urinary Pb concentration level and essential trace elements (Ca, Mg, Zn and Fe) between ASD children and TD children according to children’s age (in months). The data of the concentration level of the elements was not normally distributed. Therefore, a non-parametric test was used to analyse the essential trace element data. The outliers were retained, and log transformation was not performed to preserve the true findings. The general characteristics of the ASD children and TD children are shown in Table 1.

**Table 1:** Respondents characteristics and urinary Pb level for each variable in the study

	ASD (*n* = 81)				TD (*n* = 74)				Chi square test variables versus [A and B]	
Variables	*n* (percentage) [A]	Urinary Pb mean ± Std Dev (μg/dL)	95% CI (μg/dL)	*p*-value †	*n* (percentage) [B]	Urinary Pb mean ± Std Dev (μg/dL)	95% CI (μg/dL)	*p*-value †	*x*^2^-value	*p*-value
Parent’s background										
Age (years)	36.74 ± 3.88 ^#^				35.20 ± 5.45 ^#^				−2.393 ^ℇ^	0.017*
Age group										
30 years old and below	1 (1.20)	0.17 ± 0.00	-	0.593	14 (18.90)	0.59 ± 0.41	0.35, 0.83	0.720	13.836	***<***0.001*
More than 30 years old	80 (98.80)	0.26 ± 0.32	0.19, 0.33		60 (81.10)	0.58 ± 0.41	0.47, 0.69			
Gender										
Male	19 (23.50)	0.24 ± 0.18	0.15, 0.32	0.648	22 (29.70)	0.53 ± 0.41	0.35, 0.72	0.428	0.782	0.376
Female	62 (76.50)	0.27 ± 0.35	0.18, 0.36		52 (70.30)	0.60 ± 0.41	0.49, 0.72			
Education level										
Primary education	0 (0.00)	-	-	0.064	0 (0.00)	-	-	0.083	35.187	<0.001*
Secondary education	12 (14.80)	0.18 ± 0.09	0.12, 0.23		45 (60.80)	0.66 ± 0.46	0.52, 0.79			
Tertiary education	69 (85.20)	0.28 ± 0.36	0.20, 0.36		29 (39.20)	0.47 ± 0.28	0.36, 0.58			
Monthly income (RM)	6699.94 ± 3856.52 ^#^				3960.03 ± 2025.19 ^#^				−4.867 ^ℇ^	<0.001*
Income classification										
B40	39 (48.10)	0.29 ± 0.41	0.16, 0.43	0.761	58 (78.40)	0.61 ± 0.44	0.50, 0.73	0.379	17.922	<0.001*
M40	34 (42.00)	0.24 ± 0.19	0.17, 0.31		16 (21.60)	0.47 ± 0.26	0.33, 0.61			
T20	8 (9.90)	0.22 ± 0.06	0.16, 0.27		0 (0.00)	-	-			
Residential area										
Kuala Lumpur	31 (38.30)	0.24 ± 0.14	0.19, 0.29	0.387	61 (82.40)	0.60 ± 0.42	0.49, 0.70	0.402	31.260	<0.001*
Outside Kuala Lumpur	50 (61.70)	0.28 ± 0.38	0.17, 0.39		13 (17.60)	0.52 ± 0.38	0.29, 0.75			
Children’ background										
Age (years)	5.63 ± 0.60 ^#^				5.45 ± 0.83 ^#^				−1.163 ^ℇ^	0.245
Age group										
4 years old and below	5 (6.20)	0.81 ± 1.02	−0.46, 2.07	0.056	10 (13.50)	0.36 ± 0.15	0.25, 0.46	0.068	2.384	0.123
More than 4 years old	76 (93.80)	0.23 ± 0.17	0.19, 0.27		64 (86.50)	0.62 ± 0.43	0.51, 0.72			
Gender										
Male	68 (84.0)	0.27 ± 0.33	0.19, 0.35	0.887	39 (52.70)	0.65 ± 0.47	0.50, 0.80	0.205	17.663	<0.001*
Female	13 (16.00)	0.24 ± 0.19	0.13, 0.35		35 (47.30)	0.50 ± 0.32	0.39, 0.61			
Race										
Malay	63 (77.80)	0.28 ± 0.35	0.19, 0.37	0.807	70 (94.60)	0.57 ± 0.41	0.47, 0.67	0.102	8.980	0.003*
Non-Malay	18 (22.20)	0.21 ± 0.07	0.18, 0.25		4 (5.40)	0.80 ± 0.33	0.27, 1.33			
Immunisation status										
Up to date	80 (98.80)	0.26 ± 0.32	0.19, 0.33	0.369	70 (94.60)	0.58 ± 0.42	0.48, 0.68	0.756	1.026	0.311
Missed	1 (1.20)	0.26 ± 0.00	-		4 (5.40)	0.58 ± 0.26	0.17, 1.00			
BMI	16.28 ± 3.13 ^#^				15.71 ± 3.70 ^#^				−1.739 ^ℇ^	0.082
Speak at 3 years old										
Yes	14 (17.30)	0.18 ± 0.56	0.15, 0.21	0.240	74 (100.00)	0.58 ± 0.41	0.49, 0.68	-	107.813	<0.001*
No	67 (82.70)	0.28 ± 0.34	0.20, 0.36		0 (0.00)	-	-			
ASD among siblings										
Yes	7 (8.60)	0.16 ± 0.04	0.13, 0.20	0.118	4 (5.40)	0.50 ± 0.31	0.00, 1.01	0.774	0.614	0.433
No	74 (91.40)	0.27 ± 0.33	0.20, 0.35		70 (94.60)	0.59 ± 0.42	0.49, 0.69			
Maternal obstetric background										
Maternal age at pregnancy	29.99 ± 3.41^#^				29.30 ± 4.82 ^#^				−1.106 ^ℇ^	0.269
Advanced maternal age										
35 years old and below	78 (96.30)	0.26 ± 0.32	0.19, 0.33	0.484	70 (94.60)	0.58 ± 0.42	0.48, 0.68	0.293	0.015	0.903
More than 35 years old	3 (3.70)	0.28 ± 0.15	−0.09, 0.64		4 (5.40)	0.65 ± 0.15	0.41, 0.90			
Birth order										
First child	46 (56.80)	0.31 ± 0.41	0.19, 0.44	0.448	22 (29.70)	0.53 ± 0.37	0.37, 0.70	0.574	11.500	<0.001*
Subsequent child	35 (43.20)	0.20 ± 0.07	0.17, 0.21		52 (70.30)	0.60 ± 0.42	0.49, 0.72			
GDM										
Yes	17 (21.00)	0.21 ± 0.07	0.17, 0.25	0.853	19 (25.70)	0.58 ± 0.43	0.37, 0.79	0.887	0.477	0.490
No	64 (79.00)	0.28 ± 0.35	0.19, 0.37		55 (74.30)	0.58 ± 0.41	0.47, 0.69			
PIH										
Yes	2 (2.50)	0.49 ± 0.49	−3.93, 4.90	0.738	3 (4.10)	0.38 ± 0.09	0.17, 0.59	0.661	0.011	0.918
No	79 (97.50)	0.26 ± 0.31	0.19, 0.33		71 (95.90)	0.59 ± 0.42	0.49, 0.69			
Anaemia in pregnancy										
Yes	27 (33.30)	0.29 ± 0.26	0.18, 0.39	0.471	19 (25.70)	0.43 ± 0.27	0.30, 0.56	0.080	1.087	0.297
No	54 (66.70)	0.25 ± 0.34	0.16, 0.34		55 (74.30)	0.63 ± 0.44	0.52, 0.75			
Hb level at 36 weeks gestation	11.55 ± 1.22 ^#^				11.84 ± 2.89 ^#^				−0.070 ^ℇ^	0.944
Other comorbidity during pregnancy										
Yes	12 (14.80)	0.28 ± 0.20	0.15, 0.41	0.449	8 (10.80)	0.46 ± 0.34	0.17, 0.74	0.334	0.552	0.458
No	69 (85.20)	0.26 ± 0.33	0.18, 0.34		66 (89.20)	0.60 ± 0.42	0.50, 0.70			
Gestational age at birth (week)	37.99 ± 2.51 ^#^				38.26 ± 1.69 ^#^				−0.137 ^ℇ^	0.891
Prematurity										
Yes	9 (11.10)	0.26 ± 0.22	0.10, 0.43	0.674	11 (14.90)	0.72 ± 0.30	0.52, 0.93	0.056	0.485	0.486
No	72 (88.90)	0.26 ± 0.32	0.19, 0.34		63 (85.10)	0.56 ± 0.42	0.45, 0.66			
Place of birth										
Government hospital	42 (51.90)	0.25 ± 0.37	0.13, 0.36	0.046*	63 (85.10)	0.60 ± 0.43	0.50, 0.71	0.284	19.604	<0.001*
Private hospital	39 (48.10)	0.28 ± 0.24	0.21, 0.36		11 (14.90)	0.46 ± 0.28	0.28, 0.65			
State of birth										
Kuala Lumpur	30 (37.00)	0.24 ± 0.14	0.18, 0.29	0.544	42 (56.80)	0.51 ± 0.29	0.42, 0.60	0.261	6.046	0.014*
Outside Kuala Lumpur	51 (63.00)	0.28 ± 0.38	0.17, 0.39		32 (43.20)	0.68 ± 0.51	0.49, 0.86			
Mode of delivery										
Spontaneous vertex	46 (56.80)	0.25 ± 0.36	0.15, 0.36	0.309	50 (67.60)	0.57 ± 0.37	0.47, 0.68	0.924	4.631	0.099
Assisted delivery	11 (13.60)	0.27 ± 0.33	0.05, 0.50		3 (4.10)	0.47 ± 0.21	−0.07, 1.00			
Caesarean section	24 (29.60)	0.28 ± 0.21	0.19, 0.37		21 (28.40)	0.62 ± 0.53	0.38, 0.86			
Birth weight (g)	3.00 ± 0.47 ^#^				3.06 ± 0.59 ^#^				−0.658 ^ℇ^	0.512
Birth weight										
2500 g and below	8 (9.90)	0.19 ± 0.07	0.13, 0.25	0.517	9 (12.20)	0.60 ± 0.41	0.28, 0.91	0.250	0.446	0.800
2501 g until 4000 g	71 (87.70)	0.27 ± 0.33	0.20, 0.35		64 (86.50)	0.56 ± 0.39	0.46, 0.66			
More than 4000 g	2 (2.50)	0.16 ± 0.03	−0.11, 0.42		1 (1.40)	1.80	-			
Neonatal complication										
Yes	14 (17.30)	0.27 ± 0.22	0.14, 0.39	0.352	9 (12.20)	0.61 ± 0.48	0.24, 0.98	0.993	0.803	0.370
No	67 (82.70)	0.26 ± 0.33	0.18, 0.34		65 (87.80)	0.58 ± 0.40	0.48, 0.68			
Breastfeeding										
Yes	79 (97.50)	0.26 ± 0.32	0.19, 0.33	0.294	73 (98.60)	0.59 ± 0.41	0.49, 0.68	0.153	0.000	1.000
No	2 (2.50)	0.24 ± 0.00	0.22, 0.26		1 (1.40)	0.20 ± 0.00	-			
Duration of breast feeding (months)	14.68 ± 12.19 ^#^				14.70 ± 11.17 ^#^				−0.138 ^ℇ^	0.890
Duration of breast feeding										
6 months and below	28 (34.60)	0.40 ± 0.50	0.20, 0.59	0.013*	28 (37.80)	0.54 ± 0.35	0.40, 0.67	0.617	0.338	0.845
7 months until 24 months	41 (50.60)	0.20 ± 0.09	0.17, 0.22		34 (45.90)	0.57 ± 0.40	0.43, 0.71			
More than 24 months	12 (14.80)	0.19 ± 0.06	0.15, 0.22		12 (16.20)	0.72 ± 0.55	0.37, 1.07			
Environmental exposure background										
Type of the house										
Bungalow house	5 (6.20)	0.34 ± 0.36	−0.10, 0.79	0.569	5 (6.80)	0.52 ± 0.23	0.23, 0.81	0.660	24.275	<0.001*
Semi-detached house	5 (6.20)	0.24 ± 0.08	0.14, 0.35		1 (1.40)	0.81 ± 0.00	-			
Terrace house	26 (32.10)	0.20 ± 0.08	0.16, 0.23		18 (24.30)	0.63 ± 0.41	0.43, 0.84			
Condominium	26 (32.10)	0.28 ± 0.24	0.18, 0.37		7 (9.50)	0.41 ± 0.31	0.13, 0.70			
Apartment	2 (2.50)	0.16 ± 0.01	0.03, 0.30		3 (4.10)	0.41 ± 0.19	−0.07, 0.89			
Flat house	17 (29.80)	0.34 ± 0.58	0.04, 0.64		40 (54.10)	0.61 ± 0.46	0.46, 0.75			
Age of the house	22.28 ± 14.62 ^#^				18.28 ± 9.78 ^#^				−1.235 ^ℇ^	0.217
Age of the house										
25 years and below	64 (79.00)	0.27 ± 0.34	0.18, 0.35	0.535	47 (63.50)	0.54 ± 0.36	0.44, 0.65	0.593	8.746	0.013*
26-45 years	17 (21.00)	0.25 ± 0.17	0.17, 0.34		23 (31.10)	0.62 ± 0.46	0.42, 0.82			
More than 45 years	0 (0.00)	-	-		4 (5.40)	0.79 ± 0.69	−0.30, 1.89			
House nearby the main road										
Yes	48 (59.30)	0.23 ± 0.19	0.17, 0.28	0.122	41 (55.40)	0.55 ± 0.41	0.42, 0.68	0.375	0.235	0.628
No	33 (40.70)	0.32 ± 0.43	0.16, 0.47		33 (44.60)	0.62 ± 0.41	0.48, 0.77			
House nearby the factory										
Yes	9 (11.10)	0.17 ± 0.06	0.12, 0.22	0.256	11 (14.90)	0.81 ± 0.52	0.46, 1.16	0.106	0.485	0.486
No	72 (88.90)	0.27 ± 0.33	0.20, 0.35		63 (85.10)	0.54 ± 0.38	0.45, 0.64			
House nearby construction site										
Yes	15 (18.50)	0.20 ± 0.09	0.15, 0.25	0.614	18 (24.30)	0.43 ± 0.38	0.24, 0.62	0.038*	0.778	0.378
No	66 (81.50)	0.28 ± 0.34	0.19, 0.36		56 (75.70)	0.63 ± 0.41	0.52, 0.74			
Parental smoking status										
Active smoker	12 (14.80)	0.18 ± 0.08	0.13, 0.23	0.201	11 (14.90)	0.48 ± 0.46	0.17, 0.79	0.096	9.074	0.011*
Ex-smoker	16 (19.80)	0.23 ± 0.18	0.13, 0.32		3 (4.10)	0.31 ± 0.12	0.01, 0.60			
Non-smoker	53 (65.40)	0.29 ± 0.37	0.19, 0.40		60 (81.10)	0.62 ± 0.40	0.51, 0.72			
Parental risk at workplace										
Yes	5 (6.20)	0.21 ± 0.06	0.13, 0.29	0.724	13 (17.60)	0.59 ± 0.34	0.38, 0.80	0.604	4.892	0.027*
No	76 (93.80)	0.27 ± 0.32	0.19, 0.34		61 (82.40)	0.58 ± 0.43	0.47, 0.69			
Exposure to the soil										
Everyday	3 (3.70)	0.16 ± 0.02	0.12, 0.20	0.305	5 (6.80)	0.53 ± 0.42	0.01, 1.05	0.998	3.950	0.267
Once a week	15 (18.50)	0.24 ± 0.22	0.12, 0.36		22 (29.70)	0.58 ± 0.41	0.40, 0.76			
Once a month	33 (40.70)	0.20 ± 0.08	0.17, 0.23		26 (35.10)	0.57 ± 0.39	0.41, 0.73			
Never	30 (37.00)	0.35 ± 0.48	0.17, 0.53		21 (28.40)	0.61 ± 0.45	0.41, 0.82			
Sucking own hand										
All the time	7 (8.60)	0.21 ± 0.07	0.14, 0.28	0.979	2 (2.70)	0.85 ± 0.53	−3.87, 5.58	0.525	7.125	0.024*
Just before sleep	14 (17.30)	0.30 ± 0.33	0.11, 0.50		5 (6.80)	0.63 ± 0.67	−0.20, 1.46			
Never suck hand	60 (74.10)	0.26 ± 0.33	0.18, 0.34		67 (90.50)	0.57 ± 0.39	0.48, 0.67			
Washing hand practice										
Frequent	59 (72.80)	0.24 ± 0.20	0.19, 0.29	0.437	68 (91.90)	0.58 ± 0.41	0.48, 0.68	0.566	14.011	0.001*
Sometimes	15 (18.50)	0.22 ± 0.09	0.17, 0.26		6 (8.10)	0.65 ± 0.38	0.25, 1.05			
Seldom	7 (8.60)	0.55 ± 0.89	−0.27, 1.37		0 (0.00)	-	-			
Dietary habit of children										
Drinking water from tap water										
Yes	75 (92.60)	0.26 ± 0.32	0.19, 0.34	0.330	66 (89.20)	0.57 ± 0.41	0.47, 0.67	0.338	0.545	0.460
No	6 (7.40)	0.25 ± 0.10	0.15, 0.35		8 (10.80)	0.68 ± 0.38	0.36, 1.00			
PICA										
Yes	16 (19.80)	0.27 ± 0.27	0.12, 0.41	0.555	4 (5.40)	0.52 ± 0.18	0.23, 0.82	0.968	11.723	0.003*
No	47 (58.00)	0.21 ± 0.12	0.18, 0.21		61 (82.40)	0.58 ± 0.41	0.48, 0.69			
Not sure	18 (22.20)	0.39 ± 0.58	0.11, 0.68		9 (12.20)	0.60 ± 0.49	0.22, 0.97			
Vitamin consumption										
Yes	47 (58.00)	0.25 ± 0.22	0.19, 0.32	0.491	42 (56.80)	0.63 ± 0.45	0.49, 0.77	0.398	1.366	0.505
No	34 (42.00)	0.28 ± 0.41	0.14, 0.42		32 (43.20)	0.52 ± 0.35	0.39, 0.64			
Drinking milk										
More than 2 times per day	22 (27.20)	0.31 ± 0.51	0.09, 0.54	0.582	17 (23.00)	0.61 ± 0.45	0.37, 0.84	0.648	1.770	0.413
1 to 2 times per day	33 (40.70)	0.25 ± 0.24	0.17, 0.33		38 (51.40)	0.55 ± 0.39	0.42, 0.67			
Never drink	26(32.10)	0.24 ± 0.14	0.18, 0.29		19 (25.70)	0.63 ± 0.43	0.42, 0.84			
Eating fruits										
Every meal	10 (12.30)	0.20 ± 0.07	0.15, 0.25	0.893	17 (23.00)	0.66 ± 0.56	0.37, 0.95	0.732	18.052	<0.001*
Once a day	21 (25.90)	0.24 ± 0.15	0.17, 0.31		19 (25.70)	0.49 ± 0.32	0.33, 0.64			
Once a week	27 (33.30)	0.27 ± 0.26	0.16, 0.37		35 (47.30)	0.60 ± 0.38	0.47, 0.73			
Never eat	23 (28.40)	0.31 ± 0.50	0.09, 0.52		3 (4.10)	0.49 ± 0.18	0.06, 0.93			
Eating vegetables										
Every meal	16 (19.80)	0.24 ± 0.17	0.15, 0.33	0.177	12 (16.20)	0.46 ± 0.32	0.26, 0.66	0.210	2.628	0.453
Once a day	21 (25.90)	0.34 ± 0.52	0.11, 0.58		20 (27.00)	0.61 ± 0.46	0.39, 0.82			
Once a week	12 (14.80)	0.25 ± 0.32	0.04, 0.45		18 (24.30)	0.48 ± 0.29	0.34, 0.62			
Never eat	32 (39.50)	0.23 ± 0.16	0.17, 0.28		24 (32.40)	0.70 ± 0.46	0.51, 0.90			

*p < 0.05 indicates significant statistical result

^#^Mean ± standard deviation value of the variable

†Comparison of urinary Pb mean ± standard deviation level between each group of variable using Mann-Whitney *U* test/Kruskal-Wallis test

^ℇ^*z*-value from Mann-Whitney *U* test to determine comparison of urinary Pb mean ± standard deviation level between the ASD group and TD group for quantitative variable

A total of 155 preschool children (81 ASD children and 74 TD children) participated in the study. The male-to-female ratio was approximately 5:1 for ASD children and 1:1 for TD children (*p* < 0.001). Most of the children in both groups were Malays. For the ASD children and TD children, the Malay percentage were 75.0% and 94.6%, respectively (*p* = 0.003). Despite having ASD, approximately 17.3% of ASD children were able to talk at the age of 3 years old. In contrast, all TD children (100.0%) were able to speak at the age of 3 years old since the ability to speak was one of the control group’s inclusion criteria.

The parents of the ASD children were approximately 1 year older than the TD children’s parents (*p* = 0.047). Most parents in each group aged more than 30 years (*p* < 0.001), indicating that the parental age’s proxy during conception was appropriate. More than half of the ASD children were the first child in the family, while about 30.0% of the TD children were a subsequent child in the family (*p* < 0.001). Most parents had secondary education (*p* < 0.001) and were from B40 income group (less than RM5000.00/month) (*p* < 0.001).

A majority of the ASD children lived outside Kuala Lumpur, mainly in Selangor. On the other hand, most TD children lived in Kuala Lumpur (*p* < 0.001). Most ASD children were born in the government hospital (51.9%), outside Kuala Lumpur (63.0%) (*p* < 0.001). Meanwhile, most TD children were born in public hospitals (85.1%) in Kuala Lumpur (56.8%) (*p* = 0.014). Most ASD children stayed in middle-range houses (32.1% terrace houses and 32.1% condominiums), while most TD children stayed in flat houses (54.1%). The houses were older in the ASD group (22.28 ± 14.62 years), compared to TD children (18.28 ± 9.78 years) (*p* = 0.045).

Most of the parents were non-smoker (65.4% in the ASD children group, and 81.1% in the TD children group) (*p* = 0.011). The parents claimed no risk of Pb exposure at their workplace (93.8% in the ASD children group and 82.4% in the TD children group) (*p* = 0.027). There were no significant differences between the groups for parent’s gender, children’s age, children’s immunisation status, children’s BMI, ASD in family, obstetric risk factors, house location (nearby the main road, factory and construction site) and source of drinking water (*p* > 0.05). However, there was a significant mean difference of urinary Pb level between groups for the place of birth (*p* = 0.046) and the duration of breastfeeding (*p* = 0.013) among ASD children, and proximity of the house to the construction site (*p* = 0.038) among TD children.

**Fig. 2:**
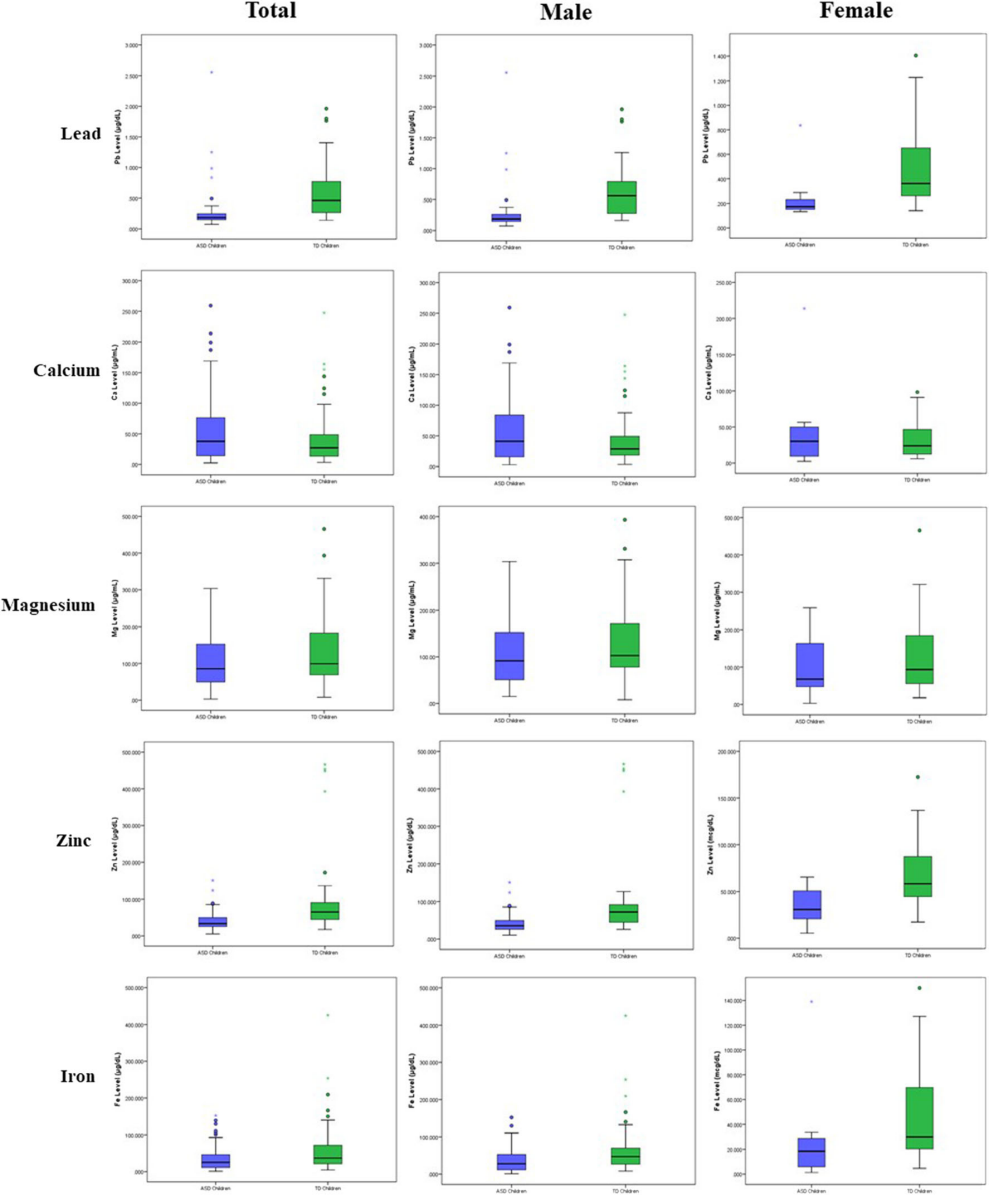
Box plots show a comparison of mean concentration level of urinary Pb and essential trace elements (Ca, Mg, Zn and Fe) between ASD children and TD children among all participants, among male children and among female children

As shown in Fig. 2 and Table 2, laboratory analysis of urinary Pb and essential trace elements revealed statistically significant differences between the groups (*p* < 0.05), except Ca (*p* = 0.096). Surprisingly, the urinary Pb levels were significantly lower in ASD children (mean 0.26 ± 0.31 μg/dL) compared to TD children (mean 0.58 ± 0.41 μg/dL) (*p* < 0.05). Urinary Mg, Zn and Fe were also lower in ASD children than TD children. Further assessment was done for all the male children and children aged above 4 years. The result revealed a similar trend for all elements. However, there was a marked difference in the mean level of elements between the groups. For children aged 4 years and below, all elements were higher in the ASD children group than the TD children group. However, the finding was not statistically significant (*p* > 0.05), except Ca (*p* = 0.002).

**Table 2:** The urinary Pb and essential trace element level between the ASD group and TD group

Urinary Pb and essential trace elements	Group	Sample	Min	Max	Mean ± Std Dev	Geometric mean	Median	IQR	Changes †	*p*-value ^#^
Overall (*n* = 155)										
Pb (μg/dL)	ASD	81	0.08	2.56	0.26 ± 0.31	0.21	0.19	0.106	+ 123%	<0.001*
	TD	74	0.14	1.96	0.58 ± 0.41	0.47	0.46	0.508		
Ca (μg/mL)	ASD	81	2.41	259.21	54.91 ± 53.88	34.04	37.71	64.93	−24%	0.134
	TD	74	3.40	247.45	41.84 ± 43.12	27.98	27.11	35.55		
Mg (μg/mL)	ASD	81	3.00	303.56	104.29 ± 72.15	76.95	85.38	103.69	+ 28%	0.060
	TD	74	7.93	465.64	133.09 ± 94.74	102.55	99.26	115.02		
Zn (μg/dL)	ASD	81	5.41	151.18	39.81 ± 24.52	33.40	33.48	24.94	+ 123%	<0.001*
	TD	74	17.39	466.26	88.88 ± 90.15	69.06	65.05	46.20		
Fe (μg/dL)	ASD	81	1.01	152.17	34.69 ± 33.28	21.33	25.27	36.75	+ 68%	0.001*
	TD	74	4.70	425.27	58.32 ± 64.26	39.18	36.86	52.17		
Among male children (*n* = 107)										
Pb (μg/dL)	ASD	68	0.08	2.56	0.27 ± 0.33	0.21	0.19	0.12	+ 141%	<0.001*
	TDC	39	0.16	1.96	0.65 ± 0.47	0.51	0.56	0.53		
Ca (μg/mL)	ASD	68	2.65	259.21	57.72 ± 53.50	37.70	41.06	70.05	−14%	0.316
	TDC	39	3.40	247.45	49.47 ± 53.46	31.51	28.58	31.52		
Mg (μg/mL)	ASD	68	14.94	303.56	106.00 ± 71.08	82.36	91.38	102.00	+ 32%	0.044*
	TDC	39	7.93	393.15	139.96 ± 90.50	111.79	102.62	96.83		
Zn (μg/dL)	ASD	68	10.56	151.18	41.13 ± 25.16	35.23	35.51	24.14	+ 156%	<0.001*
	TDC	39	25.86	466.26	105.20 ± 118.06	75.03	72.17	47.59		
Fe (μg/dL)	ASD	68	1.01	152.17	36.44 ± 32.75	23.49	27.83	42.61	+ 91%	0.005*
	TDC	39	8.14	425.57	69.56 ± 80.15	45.38	47.04	46.25		
Among female children (*n* = 48)										
Pb (μg/dL)	ASD	13	0.13	0.84	0.24 ± 0.19	0.21	0.17	0.08	+ 108%	<0.001*
	TDC	35	0.14	1.41	0.50 ± 0.32	0.35	0.36	0.40		
Ca (μg/mL)	ASD	13	2.41	213.80	40.19 ± 55.62	19.94	30.11	44.65	−17%	0.634
	TDC	35	5.99	98.29	33.35 ± 25.62	24.51	23.92	34.91		
Mg (μg/mL)	ASD	13	2.99	259.17	95.35 ± 79.97	53.92	67.74	134.83	+ 32%	0.291
	TDC	35	17.79	465.64	125.44 ± 100.00	93.14	93.57	131.49		
Zn (μg/dL)	ASD	13	5.41	65.49	32.87 ± 20.32	25.29	30.85	38.91	+ 115%	<0.001*
	TDC	35	17.39	172.50	70.70 ± 35.02	62.97	58.38	44.22		
Fe (μg/dL)	ASD	13	1.34	139.07	25.52 ± 35.92	12.87	18.50	24.65	+ 79%	0.019*
	TDC	35	4.70	149.80	45.79 ± 37.13	33.26	30.01	58.04		
Among children aged of 4 years old and below (*n* = 15)										
Pb (μg/dL)	ASD	5	0.17	2.56	0.81 ± 1.02	0.46	0.24	1.50	−56%	1.000
	TDC	10	0.19	0.60	0.36 ± 0.15	0.33	0.31	0.25		
Ca (μg/mL)	ASD	5	30.11	259.21	107.95 ± 92.77	80.13	86.03	157.42	−68%	0.019*
	TDC	10	9.11	98.29	34.18 ± 30.86	25.70	22.40	27.85		
Mg (μg/mL)	ASD	5	49.53	254.56	133.93 ± 88.58	109.69	118.69	170.55	−36%	0.371
	TDC	10	7.93	185.63	86.01 ± 53.55	65.85	82.15	95.55		
Zn (μg/dL)	ASD	5	20.82	151.18	57.22 ± 53.91	43.41	30.85	77.27	−10%	0.440
	TDC	10	25.86	82.17	51.51 ± 20.59	47.87	44.63	41.91		
Fe (μg/dL)	ASD	5	18.504	152.17	68.10 ± 54.72	51.17	55.19	98.87	−40%	0.310
	TDC	10	15.19	124.13	40.64 ± 34.89	31.78	24.68	34.02		
Among children aged of more than 4 years old (*n* = 140)										
Pb (μg/dL)	ASD	76	0.08	1.25	0.23 ± 0.17	0.20	0.18	0.10	+ 170%	<0.001*
	TDC	64	0.14	1.96	0.62 ± 0.43	0.50	0.52	0.54		
Ca (μg/mL)	ASD	76	2.41	213.80	51.42 ± 49.37	32.17	33.26	58.21	−16%	0.302
	TDC	64	3.40	247.45	43.04 ± 44.80	28.36	30.96	36.40		
Mg (μg/mL)	ASD	76	2.99	303.56	102.34 ± 71.22	75.17	84.35	104.55	+ 37%	0.019*
	TDC	64	17.79	465.64	140.45 ± 97.89	109.89	102.54	126.28		
Zn (μg/dL)	ASD	76	5.41	124.09	38.66 ± 21.56	32.83	33.60	24.51	+ 145%	<0.001*
	TDC	64	17.39	466.26	94.72 ± 95.40	73.13	71.12	48.90		
Fe (μg/dL)	ASD	76	1.01	139.07	32.49 ± 30.70	20.14	23.95	36.13	+ 88%	<0.001*
	TDC	64	4.70	425.57	61.08 ± 67.48	40.48	42.90	56.88		

*p < 0.05 indicates significant statistical result

^#^Comparison of urinary heavy metals and essential trace element mean ± standard deviation level using Mann-Whitney *U* test

†Changes in urinary heavy metal and essential trace element mean level were measured by dividing the means differences (between two groups) with the mean level of ASD group

As shown in Table 3, the overall correlation analysis demonstrated a significant positive association between the urinary Pb and the essential trace elements, except Ca (*r* = −0.01, *p* > 0.05). The association levels shown on this correlation ranged from very weak (Pb × Mg, *r* = 0.19) to moderate (Pb × Zn, *r* = 0.44). The correlation between the essential trace elements revealed a significant positive association ranging from weak (Ca × Zn, *r* = 0.25) to a very strong association (Ca × Fe, *r* = 0.87). A similar trend was found in all the male children (*n* = 107) and those aged above 4 years (*n* = 140). In the ASD and TD children groups, the correlation analysis revealed a non-significant positive association between the urinary Pb and the essential trace elements (*p* > 0.05), except for Pb × Zn (*r* = 0.26, *p* > 0.05) in male ASD children (*n* = 68).

**Table 3:**
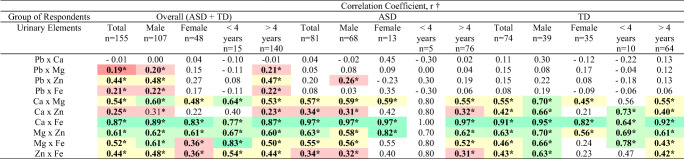
Correlation between the levels of the studied elements in the urine of the ASD group and the TD group

*p < 0.05 indicates significant statistical result

†Correlation coefficient, *r* was calculated using Spearman’s correlation test

Indicates a very weak correlation between two elements (*r* value ranges from 0.00 until 0.19), , Indicates a weak correlation between two elements (*r* value ranges from 0.20 until 0.39), , Indicates a moderate correlation between two elements (*r* value ranges from 0.40 until 0.59), , Indicates a good correlation between two elements (*r* value ranges from 0.60 until 0.79), , Indicates an excellent correlation between two elements (*r* value ranges from 0.80 until 1.00)

The overall correlation between the essential trace elements revealed a significant positive association, with the association level ranging from weak (Ca × Zn, *r* = 0.25) to a very strong association (Ca × Fe, *r* = 0.87). A similar trend was found in all the male children (*n* = 107), all children aged above 4 years old (*n* = 140), all ASD children (*n* = 81), male ASD children (*n* = 68) and ASD children aged above 4 years old (*n* = 76). The correlation between essential trace elements showed moderate to a very good association in all TD children (*n* = 74) and TD children aged above 4 years old (*n* = 64), and excellent association in male TD children (*n* = 39). The correlation between urinary Ca and Fe produced persistent good to excellent association among different groups of participants (correlation coefficient, *r* from 0.64 to 0.97). Despite the non-significant findings, the correlation between urinary Pb and Ca showed a negative very weak to weak associations among all children (*n* = 155), male ASD children (*n* = 68), ASD children aged 4 years and below (*n* = 5), female TD children (*n* = 35) and TD children aged 4 years and below (*n* = 10).

Table 4 demonstrates each urinary element’s cut-off point among the 155 children using the ROC curve analysis. The area under the curves for urinary Pb and Zn showed the significant value closest to one (0.84 and 0.81, respectively). On the other hand, the urinary Ca, Mg and Fe showed the value closest to 0.5 (0.57, 0.59 and 0.65, respectively). The cut-off point for all elements was within the standard reference level.

**Table 4:** Determination of cut-off point of each urinary elements among 155 children using ROC curve analysis and comparison with available standard references

Urinary element	Area under the curve	95% CI	*p*-value	Cut-off point ^ℇ^	Standard reference level	Lab specimen	Reference
Pb (μg/dL)	0.84	0.78, 0.91	<0.001*	0.25	5.00	Serum	ACCLPP, CDC 2012
Ca (μg/mL)	0.57	0.48, 0.66	0.134	48.64	88.00-108.00	Serum	NHANES, CDC 2011
Mg (μg/mL)	0.59	0.50, 0.68	0.060	75.87	6.00-304.00	Urine	NHANES, CDC 2014
Zn (μg/dL)	0.81	0.74, 0.87	<0.001*	65.85	100.00	Serum	ATSDR, CDC 2005
Fe (μg/dL)	0.65	0.56, 0.73	0.001*	60.24	32.00-175.00	Serum	NHANES, CDC 2008

*ACCLP* Advisory Committee on Childhood Lead Poisoning Prevention, *CDC* Centres for Disease Control, *NHANES* National Health and Nutrition Examination Survey, *ATSDR* Agency for Toxic Substances and Disease Registry

*p < 0.05 indicates significant statistical result

^ℇ^The estimation of cut-off points of elements from ROC curve analysis in the current study

Table 5 shows the multiple logistic regression analysis results of potential associated factors of ASD in both groups. Parental education, the children’s ethnicity, the children’s gender and parental smoking status were identified as significant associated factors of ASD. Parents with tertiary education had 26 times the odds of having ASD child compared to parents with secondary education (OR = 26.15, 95% CI 7.10, 96.38, *p* < 0.001). The odds of ASD in non-Malay children were 7.5 times higher than Malay children (OR = 7.52, 95% CI 1.62, 34.85, *p* = 0.010). The odds of ASD in male children were 8.5 times higher than for female children (OR = 8.52, 95% CI 2.76, 26.28, *p* < 0.001). An ex-smoker parent had 25 times of having ASD child compared to a non-smoker parent (OR = 25.29, 95% CI 4.03, 158.68, *p* = 0.001).

**Table 5:** Multiple logistic regression analysis results of associated factors for ASD

Variables	*B*	Wald	aOR	95% CI	*p*-value	*R*^2^
Parents						
Education background						
Tertiary education	3.26	24.06	26.15	7.10, 96.38	<0.001*	0.58
Secondary education			1.00			
Children						
Race						
Non-Malay	2.02	6.65	7.52	1.62, 34.85	0.010*	
Malay			1.00			
Gender						
Male	2.14	13.90	8.52	2.76, 26.28	<0.001*	
Female			1.00			
ASD among the siblings						
Yes	−0.84	0.68	0.43	0.06, 3.18	0.409	
No			1.00			
Obstetric history						
Advanced maternal Age						
More than 35 years old	0.30	0.07	1.35	0.14, 12.75	0.794	
35 years old and below			1.00			
Birth order						
First child	0.45	0.88	1.57	0.61, 4.05	0.350	
Subsequent child			1.00			
GDM						
Yes	−0.57	0.82	0.57	0.17, 1.93	0.364	
No			1.00			
PIH						
Yes	1.14	0.29	3.12	0.05, 200.93	0.592	
No			1.00			
Anaemia in pregnancy						
Yes	−0.50	0.90	0.61	0.22, 1.70	0.342	
No			1.00			
Prematurity						
Yes	0.55	0.42	1.73	0.33, 9.06	0.516	
No			1.00			
Mode of delivery		0.65			0.723	
Caesarean section	−0.49	0.63	0.62	0.19, 2.05	0.429	
Assisted delivery	0.02	0.00	1.02	0.16, 6.68	0.985	
Spontaneous vertex			1.00			
Birth weight		1.48			0.477	
2500 g and below	−1.67	0.66	0.12	0.003, 10.64	0.418	
2501 g until 4000 g	−0.88	0.16	0.42		0.688	
More than 4000 g			1.00	0.06, 30.02		
Neonatal complication						
Yes	−0.17	0.05	0.84	0.20, 3.65	0.821	
No			1.00			
Breastfeeding						
No	0.37	0.04	1.45	0.04, 59.90	0.846	
Yes			1.00			
Environmental exposure background						
Parental smoking status			12.11		0.002*	
Active smoker	1.35	2.77	3.84	0.79, 18.72	0.096	
Ex-smoker	3.23	11.88	25.29	4.03, 158.68	0.001*	
Non-smoker			1.00			
Constant	−3.21	1.17	0.04		0.280	

*aOR* adjusted odds ratio, *R*^2^ Nagelkerke *R* square

*p < 0.05 indicates significant statistical result

Table 6 shows the multiple logistic regression analysis results of urinary Pb and essential trace elements. The interactions between urinary elements Pb and Ca, Pb and Mg, Pb and Zn and Pb and Fe were significant. Therefore, these interactions were included in the multiple logistic regression analysis. The odds of ASD significantly reduced by 0.1% with increased of every 1.0 μg/dL urinary Pb after further interaction analysis (OR = 0.001, 95% CI 0.00, 0.89, *p* = 0.046). The odds of ASD significantly increased by 24.0% with increased of every 1.0 μg/dL urinary Ca (OR = 1.24, 95% CI 1.13, 1.36, *p* < 0.001). After further interaction analysis, the odds of ASD increased by only 4.0% with increased of every 1.0 μg/dL urinary Ca. However, the result was non-significant (OR = 1.24, 95% CI 1.13, 1.36, *p* < 0.001). An increment of every 1.0 μg/dL urinary Zn reduced the odds of ASD by 5.0% (OR = 0.95, 95% CI 0.91, 0.99, *p* = 0.008). However, after further interaction analysis, the odds of ASD significantly reduced by 11.0% with increment of every 1.0 μg/dL urinary Zn (OR = 0.89, 95% CI 0.83, 0.93, *p* = 0.001). An increment of every 1.0 μg/dL urinary Fe reduced the odds of ASD by 23.0% (OR = 0.77, 95% CI 0.69, 0.87, *p* <0.001). The odds of ASD reduced by only 5.0% with increment of every 1.0 μg/dL urinary Fe. However, the result was non-significant (OR = 0.95, 95% CI 0.73, 1.24, *p* = 0.698).

**Table 6:** Multiple logistic regression analysis results of urinary Pb and essential trace elements

Urinary elements	*B*	Wald	aOR	95% CI	*p*-value	*R*^2^
Without elements’ interaction						
Pb (μg/dL)	−0.78	0.53	0.46	0.06, 3.75	0.469	0.83
Ca (μg/mL)	0.22	21.44	1.24	1.13, 1.36	<0.001*	
Mg (μg/mL)	0.00	0.32	1.00	0.99, 1.02	0.571	
Zn (μg/dL)	−0.06	6.94	0.95	0.91, 0.99	0.008*	
Fe (μg/dL)	−0.26	19.24	0.77	0.69, 0.87	<0.001*	
Constant	2.88	13.99	17.73		<0.001*	
With elements’ interaction						
Pb (μg/dL)	−7.10	3.98	0.001	0.00, 0.89	0.046*	0.88
Ca (μg/mL)	0.06	0.39	1.06	0.88, 1.27	0.531	
Mg (μg/mL)	0.02	2.58	1.03	1.00, 1.06	0.108	
Zn (μg/dL)	−0.12	11.34	0.89	0.83, 0.95	0.001*	
Fe (μg/dL)	−0.05	0.15	0.95	0.73, 1.24	0.698	
Pb × Ca	0.62	2.19	1.86	0.82, 4.23	0.139	
Pb × Mg	−0.04	1.55	0.96	0.90, 1.02	0.214	
Pb × Fe	0.12	5.83	1.13	1.02, 1.25	0.016*	
Pb × Zn	−0.86	2.01	0.43	0.13, 1.39	0.156	
Constant	5.78	11.71	323.15		0.001*	

*aOR* adjusted odds ratio, *R*^2^ Nagelkerke *R* square

*p < 0.05 indicates significant statistical result

## Discussion

### Urinary Pb as a Biomonitor to Assess Body Burden of Pb

The determination of Pb in urine is considered to reflect the absorbed Pb that has diffused from plasma and is excreted through the kidneys, which accounts for about two-thirds of total elimination [87, 88]. The urinary Pb is understood to reflect Pb exposure within the last few days to weeks [88, 89]. It also explains possible long-term Pb exposure [87, 90]. The absorbed Pb from blood is deposited into calcified tissues (e.g. bone) and can be stored for decades [91, 92]. Pb is slowly released from the calcified tissue based on the bone turnover rates, either from a compact structure (slow turnover) or from a trabecular structure (rapid turnover) [92], depending on age or intensity of exposure [93]. In addition, the continuous growth of young children indicates constant bone remodelling for skeletal development. The constant bone remodelling contributes to endogenous contamination where stored Pb in the bone is continuously released into the plasma [92, 94]. The cortical bone contributes about more than a twofold concentration of Pb excreted in the urine per day compared to trabecular bone [95].

The 24-h urine collection method has its limitations, although it has been used frequently in many clinical studies. The limitations are the method is inconvenient and may potentially contaminate the urine samples with heavy metals [96]. Several investigators suggested that the short duration of urine collection can provide sufficient information about Pb excretion [97]. For instance, Gulson et al. revealed an extremely good correlation between blood-urine pairs for isotopic Pb composition, indicating that urine can serve as an alternative for blood especially in new born infants and young children [96]. Fukui et al. suggested urine Pb to be a good alternative to blood Pb measurement on a group basis, in which the urine Pb was not adjusted by creatinine concentration [98]. In this study, the spot collection of urine for Pb measurement was chosen as it is the commonest and the most preferable biological sample in the biomonitoring studies involving children. Non-invasive samples (e.g. urine) were collected instead of invasive samples (e.g. blood) as the clinical procedures are difficult to perform on young children and create parental anxiety, which could lead to less participation and potentially result in selection bias [99, 100].

### The Concentration of Pb Below Elevated Level

The urinary Pb concentration for both groups in this study was below the elevated level of 5.0 μg/dL. The highest recorded concentration level of urinary Pb was 2.5 μg/dL. Out of the 155 participants, most children (90.0%) had urinary Pb level below 1.0 μg/dL (*n* = 135). Previously accumulated data (since the early 1990s) have provided sufficient evidence about the toxic effects of Pb occurring at low concentration level [101]. Since children are more vulnerable to Pb exposure and more likely to suffer from neurodevelopmental deficits, the importance of adverse health effects in young children cannot be underestimated [102]. Previous cohort studies have shown significant inverse associations among most or all children with BLLs below 10.0 μg/dL [36, 103, 104] and as low as 1.0–2.0 μg/dL in other cohort studies [105–107]. The available evidence suggests that the mean BLLs range between 2.0 and 4.0 μg/dL in the US and European countries [108]. Our study demonstrated a significant cut-off point of 0.25 μg/dL for urinary Pb from the ROC curve analysis, indicating a possibility of a neurotoxic effect of Pb at this level. However, the minimum level to cause the neurological effect, especially in young children, cannot be concluded from the present findings.

The low urinary Pb level in all children in this study could reflect that the Pb exposure in urban Kuala Lumpur has improved. Previous studies done in urban Kuala Lumpur showed a reducing trend of Pb level among children from 5.26 μg/dL (BLLs) in 2000 [80] to 3.40 μg/dL (BLLs) in 2007 [109]. In the current study, the Pb level further dropped to 0.42 μg/dL (urinary Pb). This comparison is valid, although different biological samples were used in those studies because the Pb concentrations in urine are generally lower by a minimum factor of 10 compared to Pb in the blood [88].

The reasons for the low Pb levels in children in Malaysia over the two decades could be due to the Malaysian government’s action to phase out Pb from gasoline since early 1998 [110]. Consequently, Pb concentration in the air reduced greatly from 1990 to 2004 [111]. The Malaysian government also formulated a series of policies, including the latest National Automotive Policy (NAP) 2020 [112], to encourage the use of alternative vehicles. Examples of these vehicles are battery electric vehicles (BEVs) and public transportation (e.g. electric bus, monorail and electric train). Additionally, Malaysia planned to regulate heavy metals (including Pb) in the ceramic ware since 2014. However, in 2020, Malaysia notified the World Trade Organization (WTO) regarding the maximum release amounts for Pb for cookware during testing to a new standard level of 0.5 mg/L [113]. This new standard supersedes the 13th schedule of the Food Regulations 1985, which stated that Pb in the leachate from packaging, appliance containers and vessels used for cooking should not exceed 2.0 mg/L [114].

The Ministry of Domestic Trade, Co-operatives and Consumerism Malaysia (MDTCC) regulates mandatory safety standards for toys intended for children aged below 14 years old. The maximum acceptable migration of Pb in paint shall not be more than 90.0 ppm [115]. Malaysia also introduced local legislative frameworks to manage the country’s overall e-waste sector. These frameworks include generation, movement, recycling and disposal based on laws and legislations relevant to scheduled waste and e-waste management in Malaysia. These laws and legislations include the Environmental Quality Act (EQA) 1974, Environmental Quality (Prescribed Premises) (Scheduled Wastes Treatment and Disposal Facilities) Regulations 1989, Environmental Quality (Prescribed Premises) (Scheduled Wastes Treatment and Disposal Facilities) Order 1989, Environmental Quality (Scheduled Wastes) Regulations 2005, Customs (Prohibition of Import) Order 2012 and Customs (Prohibition of Export) Order 2017 [116].

### Reduced Urinary Pb Concentration in ASD Children

Our results do not support an early hypothesis that high Pb in the urine was associated with ASD among preschool children in urban Kuala Lumpur. Instead, Pb concentration was significantly lower (*p* < 0.05) in the ASD children’s urine samples than TD children (0.26 μg/dL for ASD children versus 0.58 μg/dL for TD children). When we adjusted for a potential confounding factor, such as age and gender (as shown in Table 2), there was no sufficient evidence to indicate a higher urinary Pb concentration in ASD children than TD children.

However, our univariable results are consistent with several other studies since the early 1980s until 2020. These studies reported lower urinary Pb levels in ASD children than TD children, as listed in Table 7. For example, Marlowe et al. reported significantly lower Pb level in the hair sample of ASD children (mean 6.28 ± 2.12 ppm) compared to race-matched and social class-matched TD children (mean 6.66 ± 2.49 ppm) [118]. In Japan, Yasuda et al. reported significantly lower Pb level in the hair sample of ASD children (mean 0.39 ± 0.23 ppb) compared to age-matched and gender-matched TD children (mean 0.89 ± 0.50 ppb) [119]. In Turkey, Yorbik et al. reported significantly lower Pb level in the urine sample of ASD children (mean 1.19 μg/g creatinine) compared to unmatched TD children (mean 4.63 μg/g creatinine) [122]. In Saudi Arabia, Alabdali et al. reported significantly lower Pb level in the blood sample of ASD children (mean 4.73 μg/dL) compared to age-matched and gender-matched TD children (mean 6.79 μg/dL) [126]. In Jamaica, Rahbar et al. reported significantly lower Pb level in the blood sample of ASD children (mean 2.25 μg/dL) compared to age-matched and gender-matched TD children (mean 2.73 μg/dL) [127]. Five years later, the same author (Rahbar et al.) reported significantly lower Pb level in the blood sample of ASD children (geometric mean 1.92 μg/dL) compared to age-matched and gender-matched TD children (geometric mean 2.34 μg/dL) [131].

**Table 7:** List of previous studies with similar findings (reduced Pb level in ASD children compared to control group)

No.	Author	Year	Study location	Sample		Age (years)		Matched-control	Method of laboratory analysis	Type of sample (unit)	Mean ± SD lead level in case versus control		Mean difference (μg/dL)
				ASD	TD	ASD	TD				ASD	TD	
1.	Shearer et al. ^[[117^^]^	1982	USA	12	12	8.40 ± 0.60	8.00 ± 0.80	No	AAS	Hair (ppm)	3.50	4.70	−1.20
2.	Marlowe et al. ^[[118^^]^	1985	USA	28	18	8.85 ± 4.06	10.83 ± 4.55	Race and social class	AAS	Hair (ppm)	6.28 ± 2.12	6.66 ± 2.49	−0.38*
3.	Yasuda et al. ^[[119^^]^	2005	Japan	200	56	4-9	4-9	Age and gender	ICP-MS	Hair (ppb)	0.39 ± 0.23	0.89 ± 0.50	−0.50*
4.	Adams et al. ^[[120^^]^	2006	USA	51	40	3-15	3-15	Geographic location and socioeconomic status	ICP-MS	Hair (μg/g)	0.62	0.81	−0.19
5.	Kern et al. ^[[121^^]^	2007	USA	45	45	1-6	1-6	Age, gender and ethnic, vaccination status	ICP-MS	Hair ^#^	0.19 ± 0.65	0.20 ± 0.37	N/A
6.	Yorbik et al. ^[[122^^]^	2010	Turkey	30	20	3-12	3-12	No	GF-AAS	Urine (μg/g creatinine)	1.19	4.63	−3.44*
7.	Obrenovich et al. ^[[123^^]^	2011	USA	26	39	< 6	< 6	Age	ICP-MS	Hair (ppm)	0.60	0.66	−0.06
8.	Abdullah et al. ^[[124^^]^	2012	USA	22	22	9-14	9-14	Child’s gender and race, parents’ education and marital status.	LA-ICP-MS	Prenatal teeth Pb (ppm)	0.27 ± 0.27	0.38 ± 0.59	−0.11
										Postnatal teeth Pb (ppm)	0.29 ± 0.29	0.43 ± 0.61	−0.14
9.	Albizzati et al. ^[[125^^]^	2012	Italy	17	20	6-16	6-16	No	ICP-MS	Urine (ppb/mL)	0.71 ± 0.29	0.73 ± 0.29	0.02
										Hair (ppb/mL)	0.59	0.62	0.03
10.	Alabdali et al. ^[[126^^]^	2014	Saudi Arabia	30	20	3-12	3-12	No	AAS	Blood (μg/dL)	4.73	6.79	−2.06*
11.	Rahbar et al. ^[[127^^]^	2015	Jamaica	52	100	2-8	2-8	Age and gender	ICP-MS	Blood (μg/dL)	2.25	2.73	−0.48*
12.	Fuentes-Albero et al. ^[[128^^]^	2015	Spain	35	34	4–13	4–13	Age and gender	GF-AAS	Urine (ppb/mg·dL^−1^)	0.60 ± 0.19	1.32 ± 0.44	−0.72
13.	Skalny et al. ^[[129^^]^	2017	Russia	74	74	2-9	2-9	Age and gender	ICP-MS	Hair overall (μg/g)	0.51 (0.33–0.67)	0.59 (0.38–0.93)	−0.82
				35	35	2-4	2-4	Age and gender		Hair (2-4 years old) (μg/g)	0.55 (0.37–1.09)	0.57 (0.39–0.92)	−0.02
				39	39	5-9	5-9	Age and gender		Hair (5-9 years old) (μg/g)	0.45 (0.21–0.61)	0.59 (0.30–1.05)	−0.14
14.	Li et al ^[[44^^]^	2017	China	180	184	3-8	3-8	No	GF-AAS	Blood (μg/L)	56.82	58.83	−2.01
15.	Gaza et al. ^[[130^^]^	2017	Indonesia	20	20	5-17	5-17	No	ICP-MS	Hair (μg/g)	44.75 ± 0.56	48.48 ± 0.80	−3.73
16.	Rahbar et al. ^[[131^^]^	2020	Jamaica	266	266	2-8	2-8	Age and gender	ICP-MS	Blood (μg/dL)	1.92 ^a^	2.34 ^a^	−0.42*
17.	Current study	2020	Malaysia	81	74	3-6	3-6	No	ICP-MS	Urine (μg/dL)	0.26 ± 0.31	0.58 ± 0.41	−0.32*

*AAS* atomic absorption spectroscopy, *GF-AAS* graphite-furnace atomic absorption spectrometer, *ICP-MS* inductively coupled plasma mass spectrometer, *LA-ICP-MS* laser ablation inductively coupled plasma mass spectrometer, *N/A* not available

*p < 0.05 indicates significant statistical result

^#^Mean rank value based on non-parametric test

^ℇ^Standard unit for urinary elements

^a^Geometric mean

### Poor Excretory Mechanism of Pb in ASD Children

The finding of the current study also supports the value concept that the ASD children might have a decreased ability to excrete the heavy metals (including Pb) and may be considered poor detoxifiers relatively to TD children, as supported by the previous evidence [132, 133]. The decreased ability to excrete the heavy metals may lead to a higher body burden and subsequent neurological damage [121, 134]. The reason ASD children had difficulty to excrete heavy metals (including Pb) remains unclear and not well explained. However, it is postulated that the poor mechanism of Pb excretion could be explained by plausible hypotheses which are the presence of specific antioxidant in the body and competitive mechanism of Ca towards Pb during excretion.

Oxidative stress in response to environmental insults plays a role in essentially every human disease. It is also presumed to be involved in the aetiology of ASD, in which decreased antioxidant capacity and increased oxidative stress in ASD can lead to neural structure damage and impair neural functioning [135, 136]. Endogenous thiols, such as glutathione (GSH), L-cysteine, N-acetyl cysteine (NAC), taurine and melatonin, are examples of important antioxidants. These antioxidants can reduce metal availability, decrease damage to the organ cells and biological macromolecules and promote detoxification. The antioxidants work through various action mechanisms, such as scavenging free radicals, interrupting radical chain reactions and forming stable complexes with heavy metals, including Pb [121, 137]. Therefore, the decreased level of antioxidants in the body of ASD children will promote sequestration of heavy metals in the children’s brain and subsequently excrete lower concentration of heavy metals in the urine [121].

### Role of Essential Trace Element Towards Pb

Besides urinary Pb, the concentration levels of certain essential trace elements were significantly lower in ASD children than TD children; urinary Zn (39.81 μg/dL for ASD children versus 88.88 μg/dL for TD children) and urinary Fe (34.69 μg/dL for ASD children versus 58.32 μg/dL for TD children). The concentration level of urinary Mg was also lower in ASD children than TD children, but the result was non-significant. However, when we adjusted the age and gender, the mean difference of urinary Mg appeared to be significant in children aged above 4 years (102.34 μg/dL for ASD children versus 140.45 μg/dL for TD children) and male children (106.00 μg/dL for ASD children versus 139.96 μg/dL for TD children). In contrast, the result showed that the urinary Ca was significantly higher in ASD children than TD children aged 4 years and below (107.95 μg/dL for ASD children versus 34.18 μg/dL for TD children).

The univariable findings of essential trace elements in this study are consistent with the previous studies for Mg, Zn and Fe. For instance, Skalny et al. demonstrated that the Mg concentration in the hair (17.91 μg/g for ASD children versus 18.84 μg/g for TD children) and urine (108.59 μg/mL for ASD children versus 118.51 μg/mL for TD children) of ASD children was lower than the unmatched TD control. However, the findings were non-significant [138]. Priya et al. demonstrated that the Mg concentrations in hair of low functioning autism (LFA) (mean 174.02 ± 20.88 μg/g), medium functioning autism (MFA) (mean 202.21± 24.26 μg/g) and high functioning autism (HFA) (mean 236.31 ± 28.35 μg/g) children were significantly lower than the control group (mean 454.36 ± 54.52 μg/g). This finding indicated that the severity of ASD increases with the reducing Mg concentration level in the hair [139]. Strambi et al. demonstrated significantly lower plasma Mg level in ASD children (mean 2.27 ± 0.33 mg/100 mL) compared to unmatched healthy children (mean 2.51 ± 0.14 mg/100 mL) [70]. A systematic review and meta-analysis study reported significantly lower Mg levels in hair (after removal of an outlier study with effect size of −0.612, *z*-value = 2.68, *p* = 0.007) and serum (effect size of −0.105, *z*-value = 5.88, *p* < 0.001) of ASD children than healthy controls [140].

As for Zn, Priya et al. demonstrated the significantly lower concentration of Zn in the hair of LFA children (mean 130.46 ± 15.65 μg/g) than the control group (mean 171.68 ± 20.60 μg/g) [139]. Li et al. reported significantly lower concentration of Zn in the serum of ASD children (mean 78.70 ± 7.00 ng/mL) compared to age-matched and gender-matched healthy controls (mean 87.70 ± 8.70 ng/mL) [141]. Saghazadeh et al. reported a significant effect size of −0.361 (*z*-value = 2.31, *p* = 0.021), indicating that the ASD patients (*n* = 513) had lower blood Zn levels than controls (*n* = 333), after excluding two outlier studies. Further sensitivity hair sample analyses indicated that Asian patients with ASD (*n* = 236) had lower Zn levels in the hair (standardised mean difference (SMD) = −1.493, *p* = 0.002) than their Asian counterparts (*n* = 306), after excluding an outlier study [140].

As for Fe, Lubkowska et al. demonstrated that the Fe concentration in hair of ASD children (mean 9.02 ± 4.62 μg/g) was significantly lower than age-matched healthy controls (mean 10.05 ± 2.92 μg/g) [142]. Additionally, Saghazadeh et al. reported a significant effect size of −1.410 (*z*-value = 2.38, *p* = 0.017), indicating that the Fe levels in the hair of ASD children were lower than healthy children, after excluding an outlier study [140].

As for Ca, our univariate analysis contradicted with other trace elements (Mg, Zn and Fe). However, a recent study reported a similar finding, whereby the higher concentration of Ca was found in the serum of ASD children (median 109.16, 25–75 percentiles 103.55–113.5) than age-matched and gender-matched neurotypical children (median 106.71, 25–75 percentiles 103.82–112.3) [143]. However, the result was non-significant.

From the regression analysis of urinary trace elements, urinary Zn appeared to be a protective factor of ASD (OR = 0.95, 95% CI 0.91, 0.99, *p* = 0.008). The protective effect exerted by the urinary Zn was significantly increased after further interaction analysis (OR = 0.89, 95% CI 0.83, 0.95, *p* = 0.001). The urinary Fe was found to exert a protective effect towards ASD (OR = 0.77, 95% CI 0.69, 0.87, *p* < 0.001). However, the protective effect of urinary Fe was reduced and non-significant after further interaction analysis (OR = 0.95, 95% CI 0.73, 1.24, *p* = 0.698). These findings signified that the presence of essential trace elements in the body, especially Zn and Fe, is crucial to counteract Pb’s neurotoxic effect.

The essential trace elements (e.g. Mg, Zn and Fe) play a vital role as antioxidant agents, whereby the presence of these elements in the body helps to prevent redistribution and accumulation of metal in tissues, reduces metal availability, decreases toxicity, stabilises cell membranes and decreases damage to biological macromolecules [137]. These elements also decrease teratogenic toxicity by decreasing the replacement of essential ions, forming insoluble metal-mineral complexes and producing metal-binding proteins (MT) [137]. Essential trace elements also decrease gastrointestinal absorption of heavy metals and decrease its distribution through competitive absorption mechanism [137]. However, our findings failed to support the theory. The correlation between urinary Pb and essential trace elements showed a significantly positive very weak to moderate correlation coefficient (*r*-value ranged from 0.19 to 0.44), except for the correlation between urinary Pb and Ca, which showed a non-significantly negative very weak correlation (*p* > 0.05).

### Assessment of Other Associated Factors of ASD

Several associated factors of ASD were identified. These factors were ethnicity, parental education, children’s gender and parental smoking status. Our finding showed that parents with tertiary education had 26 times the odds of having an ASD child than parents with secondary education (OR = 26.15, 95% CI 7.10, 96.38, *p* < 0.001). This finding was supported by Eow et al., where the odds of having an ASD child among mother with tertiary education was 3.5 times higher compared to mother with secondary education or lower (OR = 3.47, 95% CI 1.00, 5.94) [144].

In terms of ethnicity, the proportion of non-Malay was low in the ASD children group (*n* = 18/63 (22.2%)) and TD children group (*n* = 4/70 (5.4%)). However, ethnicity (i.e. non-Malay) contributed a significant risk factor towards ASD (OR =7.52, 95% CI 1.62, 34.85, *p* = 0.010). A study reported that the non-Malay children had about 4.5 times the odds of developing ASD compared to Malay children (OR = 4.52, 95% CI 2.10, 6.94) [144].

As for gender, the male-to-female ratio of ASD children was 5:1. This ratio is higher than the previously reported ratio of 4:1 [145] and 3:1 [146, 147]. Therefore, male gender was a significant risk factor for ASD (OR = 8.52, 95% CI 2.76, 26.28, *p* < 0.001).

Lastly, the findings showed that parents (either father or mother) who were an ex-smoker had higher odds of having an ASD child than non-smoking parents (OR = 25.29, 95% CI 4.03, 158.68, *p* = 0.001). However, the finding was non-significant for parents who were an active smoker, indicating that the exposure towards heavy metals (including Pb) might occur during prenatal and antenatal periods. The parents’ decision to stop smoking might be influenced by their children being diagnosed as ASD. The risk of getting an ASD child is still high (OR = 3.53, 95% CI 1.30, 9.56) although the pregnant mother is only a second-hand smoker (mostly related to smoking husband or spouse) [148].

### Recommendation

This study focussed on the prevention strategies for ASD, particularly from environmental health and nutrition perspectives. Since there is no safe Pb level, young children should not be exposed to Pb. If they are still being exposed to Pb, the exposure level should be minimised. Top stakeholders (i.e. government) should initiate and improve preventive measures by implementing the relevant laws and legislations. The current regulations stated that the standard Pb level should be frequently revised and amended when necessary. The enforcement should be strengthened to control and monitor Pb-based product manufacturing (e.g. paint, ceramic ware, toys, electric and electronic devices).

We also recommend the government, especially the MOH, to initiate the first national Pb screening programme among newborn babies and preschool children. This practice has been done in the USA for a few decades ago. The programme could probably start by identifying the high-risk group of babies and young children through a risk-based assessment of the family’s socio-demographic background. Additionally, the prevalence of childhood exposure at the national level and burden of environmental-related disease could be identified and analysed for further action by the relevant stakeholders. The MOH could also decide on the different types of samples for biomonitoring depending on the objectives (short-term or long-term exposure monitoring) and laboratory analysis costs. At the family level, parents or caretakers should have adequate knowledge about the health effect of toxic environmental elements to minimise Pb exposure to their children. Parental knowledge about Pb exposure should be improved via various health educations, either from mass media, electronic social media and health care centre (e.g. health clinics and hospitals).

In terms of nutrition, we recommend the parents or caretakers to provide adequate essential trace elements to their children. As mentioned in this article, the essential trace elements provide many benefits to the children’s body when consumed adequately. According to the Recommended Nutrient Intakes (RNI) for Malaysia 2017 by the National Coordinating Committee on Food and Nutrition, MOH [77], the recommended Ca intake for children age 1–3 years and 4–6 years is 700.0 mg per day and 1000.0 mg per day, respectively. For Mg, the recommended intake for children age 1–3 years old and 4–8 years old is 80.0 mg per day and 130.0 mg per day, respectively. On the other hand, the recommended Zn intake for children age 1–3 years old and 4–6 years old is 4.2 mg per day and 5.2 mg per day, respectively. Lastly, the recommended Fe (with 10.0% bioavailability) intake for children age 1–6 years old is 6.0 mg per day, whereas the recommended Fe (with 15.0% bioavailability) intake for 1–6 years old is 4.0 mg per day.

### Limitations

The results of the current study should be interpreted carefully. Pb and essential trace elements were investigated only in the urine samples, which may not fully explain a complex pathological mechanism occurring in the brain due to these elements. Moreover, the relatively inconsistent results in the Pb levels of ASD children may be attributed to the heterogeneity (spectrum) of ASD, subjects’ diverse geographic locations or methodological differences. Nevertheless, these results indicated that further studies are warranted to investigate the possible role of Pb and other heavy metals in ASD.

## Conclusion

The Pb concentration level in the urine samples of both groups was below the CDC’s elevated level. The study also found that the Pb concentration level was significantly lower in ASD children than TD children. The low Pb concentration level may be due to the poor detoxifying mechanism, which retains more Pb in the body while excreting less Pb in the urine. In addition, significantly lower concentration of the essential trace elements, namely urinary Mg, Zn and Fe, may augment the neurotoxic effect of Pb in ASD children. These findings imply the importance of essential trace elements in protecting the children’s central nervous system. Prevention strategies should be consistent and involve stakeholders and parents’ participation to ensure the children’s exposure towards Pb is minimised. Prevention strategies are crucial to provide optimum nutrition to reduce the occurrence and the progress of ASD among preschool children.

## Data Availability

All data generated or analysed during this study are included in this published article. The datasets used and analysed during the current study are available from the corresponding author on reasonable request.
